# Novel Features of Cellulose-Based Films as Sustainable Alternatives for Food Packaging

**DOI:** 10.3390/polym14224968

**Published:** 2022-11-16

**Authors:** Sofia Romão, Ana Bettencourt, Isabel A. C. Ribeiro

**Affiliations:** Research Institute for Medicines (iMed.ULisboa), Faculty of Pharmacy, Universidade de Lisboa, Avenida Prof. Gama Pinto, 1649-003 Lisboa, Portugal

**Keywords:** biodegradable polymers, cellulose, cellulose derivatives, active packaging, intelligent packaging

## Abstract

Packaging plays an important role in food quality and safety, especially regarding waste and spoilage reduction. The main drawback is that the packaging industry is among the ones that is highly dependent on plastic usage. New alternatives to conventional plastic packaging such as biopolymers-based type are mandatory. Examples are cellulose films and its derivatives. These are among the most used options in the food packaging due to their unique characteristics, such as biocompatibility, environmental sustainability, low price, mechanical properties, and biodegradability. Emerging concepts such as active and intelligent packaging provides new solutions for an extending shelf-life, and it fights some limitations of cellulose films and improves the properties of the packaging. This article reviews the available cellulose polymers and derivatives that are used as sustainable alternatives for food packaging regarding their properties, characteristics, and functionalization towards active properties enhancement. In this way, several types of films that are prepared with cellulose and their derivatives, incorporating antimicrobial and antioxidant compounds, are herein described, and discussed.

## 1. Introduction

It is essential to guarantee the quality and safety of food since their loss can lead to foodborne illnesses, constituting a public health problem [1]. According to the Centers for Disease Control and Prevention (CDCP), 48 million people get sick from a foodborne illness, 128,000 people are hospitalized, and 3000 people die each year. One of the well-known causes of these diseases are biological contaminants—pathogens—such as bacteria, viruses, fungi, or parasites, with the predominant symptoms being nausea, vomiting, diarrhea, fever, and gastroenteritis [2,3].

Due to these contaminations, the food becomes susceptible to biofilm formation, a survival strategy for bacteria and fungi against adverse environmental conditions such as antimicrobial chemical agents, oxidative stress, exposure to ultraviolet rays, and pH variations [4]. Biofilms are complex microbial ecosystems that are formed by one or more species which are immersed in an extracellular polymeric matrix of different compositions depending on the type of food manufacturing environment and the colonizing species [1,5]. Biofilms represent a major concern for the food industry. Bacteria adhere to food or packaging surfaces, develop organized structures and later, they may be responsible for foodborne diseases. Once they have developed, biofilms are difficult to eradicate, mainly due to their extremely strong and stable matrix [6]. In this way, biofilms may be a source of contamination, causing food spoilage, and consequently, food waste.

According to the Food and Agriculture Organization of the United Nations (FAO) (2011), “food waste corresponds to the loss of quantity and quality of food throughout the food chain, from production, post-harvest, to processing”. Every year, around 1.3 billion tons are wasted around the world, which is equivalent to a third of the food resources that are produced for human consumption [7].

Several factors may lead to food waste: errors in food production; contamination from the equipment that is used at each stage of the chain; the presence of specific contaminants above the limits that were established by the regulators; the discharge of food that does not detain the optimal color, shape, or size; food that is not consumed as it is outdated. Nevertheless, the factor that will have a higher impact is overproduction [8].

**Figure 1:**
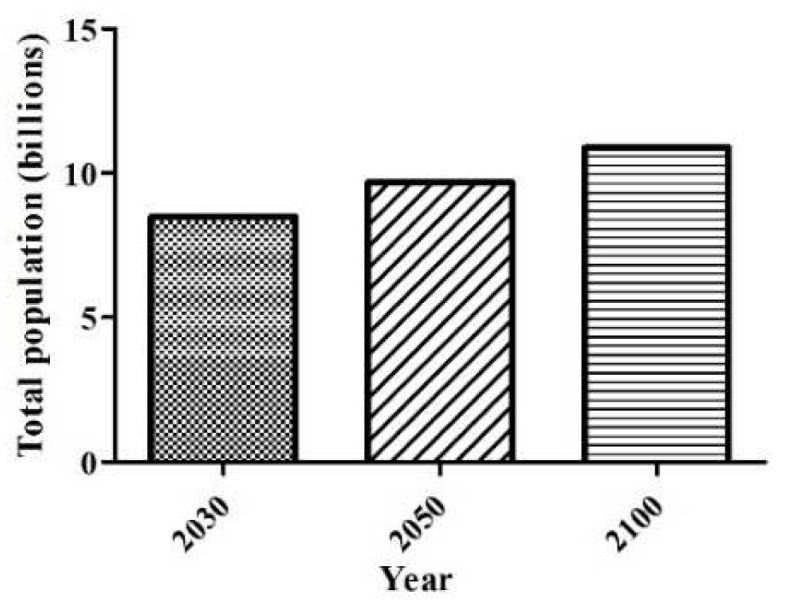
Estimative of total world population up to 2100.

According to the 2022 data, the estimate is to reach 8 billion on 15 November 2022, and the average projection indicates that the global population will continue to grow to around 8.5 billion in 2030, 9.7 billion in 2050, and 10.4 billion in 2100 (Figure 1) [9].

To keep up with population growth, an increase in food production of around 70% is expected. Thus, it is to be expected that there will be more and more food waste, having a significant negative impact on the economic (loss to farmers, retailers, and consumers), social (supply for a growing population may not be guaranteed), and environmental aspects (ground, water, energy implications, and greenhouse gas emissions) [10,11].

Packaging separates the products from the external settings and has usually the purposes of containment, protection, convenience, and communication [12]. The use of food packaging can reduce food waste, food contamination, and related foodborne illnesses. This can be achieved if the packaging protects against odors, dust, microorganisms, gases, insects, and radiation/light [13]. The packaging must also be impermeable and non-toxic, with it aiming to provide useful information to the consumers. In this way, packaging increases the shelf life of the food and assures its quality and safety [14]. Nevertheless, the food packaging industry is one of several industries that uses plastic the most, accounting for 40.5% of its use [15]. Plastic is an issue that has received special attention due to its abundance, non-degradable nature, and persistence, as well as its impacts on biodiversity and the environment [16]. In 2020, the global production of plastic reached almost 370 million tons, with a decrease of 1 million tons from 2019 to 2020. Due to the COVID crisis, the European plastics value chain, which is composed by plastics producers, plastics converters, plastics recyclers, and machinery manufacturers, experienced a decrease both in its production and demand levels. In Europe, it reached 55 million tons in 2020, corresponding to a decrease of 2 million tons, when it was compared to that in 2019 [15].

The purpose of packaging is to protect the food from contamination, and any physical damage during transport, to preserve it from undesirable chemical and atmospheric environments, and in this way, to maintain its quality, a prolonged shelf life, and to avoid wasting the food products. Packaging is essential for the distribution process of the food industries, with the purpose of delivering their products into the market without them being damaged [17]. In package production, different polymers are used. Polymers are original, lightweight, easily processed and molded, and have multiple physical, mechanical, and optical characteristics, making them effective in meeting the requirements of effective packaging [18].

Most of the polymers that are used for packaging production can be obtained from petroleum, however, their disposal and time for degradation are a matter of great concern regarding environmental, health, and economical issues. Thus, the food packaging sector feels the need to be constantly innovating and trying to discover new alternatives [19].

## 2. Biopolymers Usage as Food Packaging Alternatives

**Figure 2:**
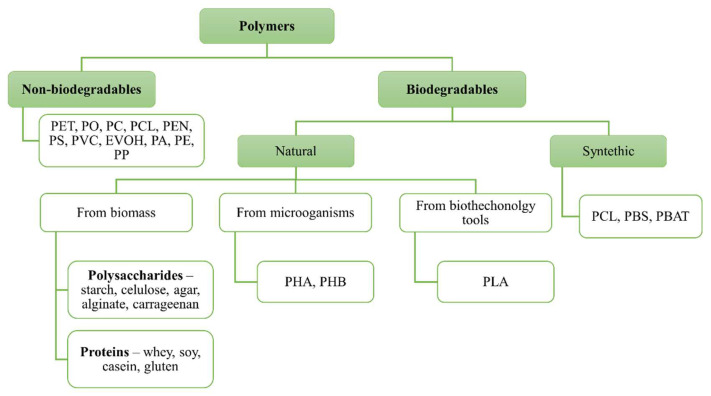
Classification of polymers proposed for food packaging.

Food packaging polymers can be classified as non-biodegradable and biodegradable (Figure 2). The non-biodegradable polymers are those that are based on petroleum such as polyolefins (PO) and polyesters, polyethylene terephthalate (PET), polycarbonate (PC), and polyethylene naphthalate (PEN). The other prominent materials are polystyrene (PS), polyvinyl chloride (PVC), ethylene vinyl alcohol (EVOH), polyamide (PA), polyethylene (PE), and polypropylene (PP). The biodegradable polymers can be of natural or synthetic origins (Figure 2). The natural polymers are obtained from biomass products (polysaccharides—starch, cellulose, agar, alginate, and carrageenan; proteins—whey, soy, casein, and gluten; lipids), from microorganisms (polyhydroxyalkanoate (PHA) and polyhydroxybutyrate (PHB)), and through biotechnology tools (polylactide and polylactic acid (PLA)). Some examples of biodegradable synthetic polymers are aliphatic polyester (polycaprolactone (PCL)), aliphatic copolyesters (polybutylene succinate (PBS)), and aromatic copolyesters (polypolybutyrate adipate terephthalate (PBAT)) [14,17,18].

Different alternative polymers have been gaining more attention as their use is considered to be important for the development of eco-friendly materials. As alternatives to the use of conventional plastic packaging, biological-based packaging has emerged, with biodegradable polymers, which have benefits for the environment [17,18]. Some of the polymers that have been proposed will be further presented.

### 2.1. Biobased Polymers

**Figure 3:**
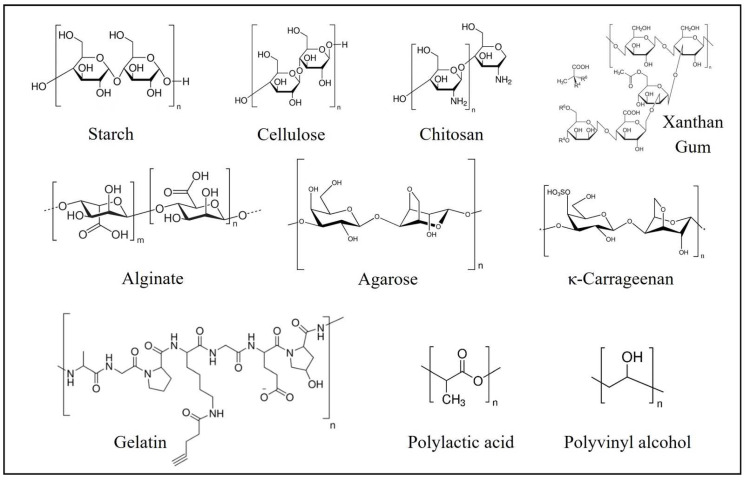
Molecular structure of various biopolymers used in food packaging.

There are many examples of biopolymers used in food packaging (Figure 3).

Among the biobased materials, poly(lactic acid) (PLA) has emerged as the most favored bioplastic. PLA is an aliphatic polyester that can be produced by the polymerization of lactic acid (LA) which is derived from agricultural feedstocks. It is non-toxic, exhibits a suitable tensile strength, and it has greater thermal processability, which distinguishes it from other plastics. It exists in three isomeric forms, poly(L-lactide) (PLLA) and poly(D-lactic acid) which are crystalline polymers, and poly(D,L-lactide) that presents 50% of D and 50% of L, thus leading to it being an amorphous polymer. PLA has some limitations such as a low heat stability and a low barrier capability. The emerging research is exploring different approaches to overcome the mentioned drawbacks, namely using reinforced fillers and blending them with other polymers [20].

Natural polymers are the most promising since they are abundantly available in nature in the form of biomass, and most of them can be obtained from agricultural resources. Polysaccharides are the most important and popular bio-based polymers that are used to make bioplastics [21]. 

For instance, starch is hugely accessible, and it has a low cost, biodegradable features, and non-toxic properties [14]. It is a polysaccharide that is mostly found in carbohydrate plants, and it consists of a large number of glucose units that are linked by 1,4- and 1,6-glycosidic bonds, leading to linear and helical structures of amylose (amorphous and crystalline phases) and a branching structure of amylopectin (amorphous phase), respectively [19]. Depending on the source, starch can present significant differences regarding its properties. The world production of starch is predominantly based on four raw materials: corn, wheat, cassava, and potatoes. Although the starch from these sources is used in food packaging industry, it has unfavorable physical properties, thus making it unsuitable for this purpose. To overcome this issue, starch is functionalized with different plasticizers to have deformable thermoplastic properties—thermoplastic starches (TPSs). It has been very promising among the biobased materials that are available for the production of biodegradable food packaging [22].

Another promising natural polymer is chitosan that is a linear amino polysaccharide which is composed by D-glucosamine and N-acetyl-D-glucosamine units that derive from chitin after deacetylation. It retains excellent film forming properties and an antimicrobial activity, it is non-toxic, biocompatible and biodegradable, and in addition to this, it also presents a chelation ability. Chitosan has some solubility in acetic acid and hydrochloric acid, which leads to the film-forming ability. Chitosan-based films can be fabricated by casting methods, coating, layer-by-layer assembly, and they have modified characteristics such as antimicrobial, antioxidant, mechanical, and barrier properties. These films have been applied to meat, fruit, and vegetables due to their preservation properties and they can be used in the form of pure chitosan films, chitosan/biopolymer films, chitosan/synthetic polymer films, or chitosan derivative films [23,24].

Carrageenan, which is a polysaccharide that is extracted from marine algae, is one of the most promising phycocolloids that demonstrates an excellent film-forming ability. Carrageenan is obtained from red seaweeds of the class *Rhodophyceae*, and its polymer chains are formed by alternate units of D-galactose and 3,6-anhydrogalactose that are joined by α-1,3- and β-1,4-glycosidic linkages. The sulfate groups on the disaccharide repeating unit determine its classification into one of three major carrageenan types: lambda (λ), kappa (κ), and iota (ι). κ-Carrageenan has the strongest gelling ability. Using carrageenan for edible films and coatings covers a wide range of food industry applications such as fresh and frozen fish, meat, dried food product, and fruit coating. Moreover, carrageenan can also be functionalized to improve its properties, particularly in active packaging applications [25].

Another promising polysaccharide is cellulose. Cellulose is particularly special since it is a highly available, renewable, and biocompatible raw material [26]. It is composed of thousands of repeating D-glucose units that are linked by β-1,4 glycosidic bonds, and it is characterized by its hydrophilicity and chemically modifying capacity. Cellulose could act as a food filler or stabilizer, which can improve the stability, texture, and sensory characteristics of the food matrices [27]. Cellulose and its derivatives are the most abundantly used polymers in the food packaging. Cellulose can be obtained from the cell walls of plants [28]. Owing to their unique characteristics, such as biocompatibility, environmental sustainability, low price, mechanical properties, and biodegradability, cellulose and its derivatives are receiving a lot of attention in the food packaging. In addition, cellulose has a high thermal resistance, the ability to carry antioxidant and antibacterial agents, it can act as a barrier against ultraviolet rays, and it is non-toxic. In this way, it contributes to increasing the nutritional value and the shelf life of fruits and vegetables. However, they also have many limitations, like a high water absorption capacity and perform insufficient interfacial adhesion [28,29,30].

Cellulose and its derivatives present suitable and interesting properties for the production of food packaging biodegradable alternatives, and those will be further addressed.

### 2.2. Cellulose Derivatives and Properties

**Figure 4:**
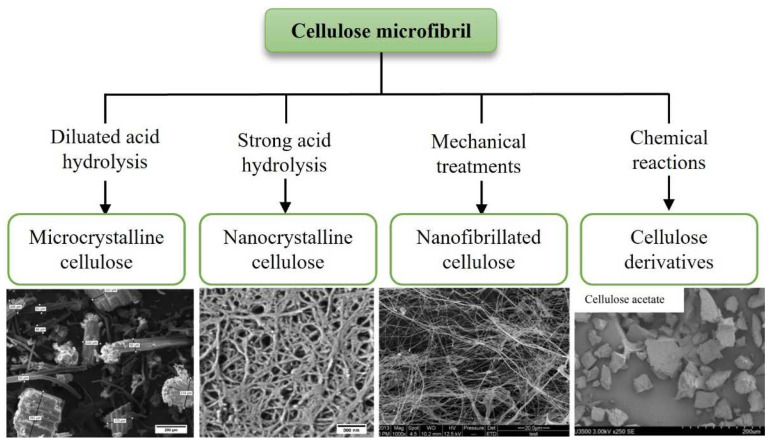
Examples of different cellulose types, according to different preparation methods. Surface electron microscopy images illustrating different microstructures; these were reproduced with permission. [31,32,33]. (Copyright 2016, 2020, 2022, Elsevier.)

According to the type of treatment that cellulose sources are submitted to, namely acid hydrolysis, and other chemical reactions or mechanical treatments, cellulose can be classified in different shapes and sizes, corresponding to different microstructures features and properties such as nanocrystalline cellulose (NCC), nanofibrillated cellulose (NFC), and microcrystalline cellulose (MCC) (Figure 4) [27].

When cellulose undergoes diluted acid hydrolysis, MCC is originated, and rod-like particles are formed at a microscale diameter and length. The obtained MCC shows high crystallinity since the amorphous regions of the cellulose chain are partially removed during this type of treatment [34]. On the other hand, from strong acid hydrolysis, NCC is obtained, while from mechanical treatments, such as high-pressure homogenization or the application of high-intensity ultrasounds, NFC is generated. These two types of cellulose are classified as a nano-cellulose because of their nanoscale dimensions [35].

**Figure 5:**
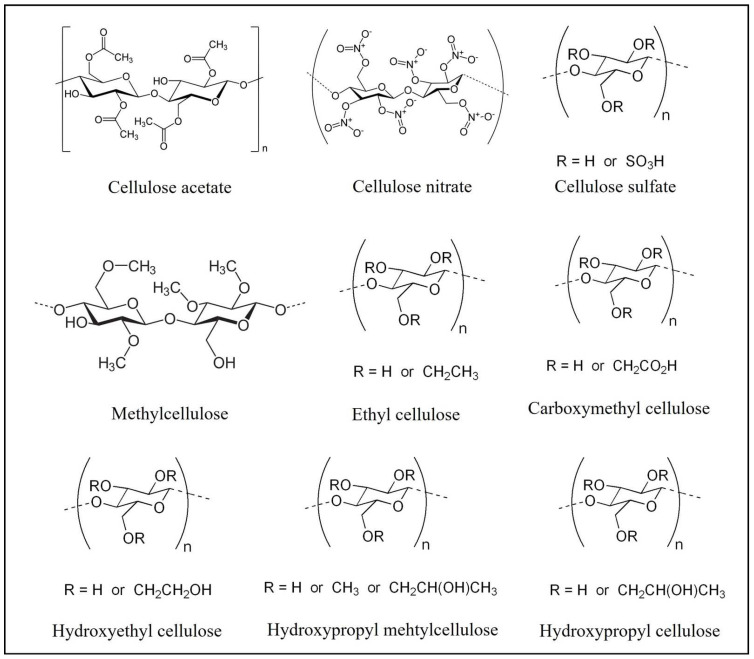
Examples of cellulose derivatives and respective molecular structure.

The cellulose derivatives include various esters and ethers, such as cellulose acetate (CA), cellulose nitrate (CN), cellulose sulfate (CS), ethyl cellulose (EC), methylcellulose (MC), carboxymethyl cellulose (CMC), hydroxyethyl cellulose (HEC), hydroxypropyl cellulose (HPC), hydroxypropyl methylcellulose (HPMC), and others (Figure 5) [27]. These will be further discussed, as we consider their importance, properties, characteristics, functionalization, and applications.

#### 2.2.1. Cellulose Acetate

Cellulose acetate (CA) is an ester that is present in green plant cell walls, and it was first discovered in 1865 by Schutzenberger as a thermoplastic biodegradable polymer. CA is obtained by the reaction of cellulose with acetic anhydride and acetic acid in the presence of sulfuric acid. This derivative is insoluble in water, and it is soluble in acetone, dioxane, methyl acetate, and acetic acid. However, the solubility of CA depends on the degree of substitution [36]. It has stable hydrolytic capabilities, is decomposable, and is non-toxic. It presents a relatively low cost, is heat resistant, shows an excellent film-forming property, and a high chemical and mechanical stability [37]. As a membrane, CA is widely used in wastewater treatment [38], gas separation [39], energy generation [40], water desalination [41], wound dressing [42], biomedical purposes [43], and in the food and pharmaceutical industries [44]. For instance, in Said Mahmoud et al.’s work, a CA/nano Zero Valent Iron (nZVI) was indicated to efficiently remove different contaminants from domestic wastewater at a 60% water content because the nZVI improved the mechanical properties of the membrane such as oil and organic solvents resistance [45]. When it is used as fibers or nanofibers, CA can act in tissue engineering scaffolds, transdermal delivery systems, wound dressings, postsurgical devices, and implants. As an example, a study demonstrated two functionalities of CA nanofiber, i.e., its antibacterial properties and its slow release of vitamins by impregnating zinc oxide nanoparticles and vitamins (B_2_ and C). The nanoparticles revealed its antibacterial activity (>99%) against *Staphylococcus aureus* and *Escherichia coli*, and thus, its ability to avoid infections. On the other hand, the slow release of vitamins showed the potential of it as an oral delivery system [46]. In the food packaging field, polylactic acid/cellulose acetate films were fabricated via solvent casting using phenyl salicylate as an ultraviolet absorbent and antibacterial agent. The results showed that the films presented a complete absorption effect in the region of UV-C (280–100 nm) and UV-B (315–280 nm) and more than 95% absorption in the region of UV-A (400–315 nm). Moreover, the steam resistance, mechanical properties, and thermal stability of the films were improved, and they exhibited also better antibacterial activity against *E. coli*. These films were tested in fresh lilies, and this extended their storage time [47]. In another study, CA/geranyl acetate films were produced and showed excellent antibacterial activity against Gram-positive and Gram-negative bacteria, as well as good antifungal activity. Thus, these films seem promising to be used to produce active food packaging [48]. Laroque et al. prepared CA films by incorporating carvacrol in the solvent casting technique, and the results showed that carvacrol decreased the melting and glass transition temperatures of the films. The films were bactericidal against *Weissella viridescens* and *Pseudomonas fluorescens,* and this extended the shelf life of the vacuum-packaged hum by 2.8 times [49].

#### 2.2.2. Cellulose Sulfate

Cellulose sulfate (CS) is another cellulose ester that has a simple chain structure and unique biological properties. It can be obtained by sulfation procedures using solvents such as sulfuric acid, sulfur trioxide, or chlorosulfonic acid. These synthetic procedures can be heterogeneous, quasi-homogeneous, and homogeneous based on the solubility of the substrates in the reaction media [50]. Cellulose sulfate shows antibacterial, antiviral, and anticoagulant properties, and it is soluble in water at high concentrations. These properties can be related to the presence of the sulfate groups and the broad degree of substitution. CS has some advantages such as easy preparation process, an affordable cost, an excellent biocompatibility, a film-forming ability, and biodegradability [51]. CS can be used to encapsulate enzymes and cells as inhibitors for HIV viruses and anticoagulant effectors for contraception and in drug delivery [52,53]. For instance, cellulose sulfate-chitosan hydrochloride microcapsules seem to be promising drug vehicles [54].

In the food packaging field, there are not many studies on CS, but its properties make it suitable to be used in this area. In Chen et al.’s study, the cellulose sulfate-based edible films were capable of prolonging the shelf life of bananas, and in general, the films could be applied in fruit coating, soluble packaging, and oily food packaging uses [55]. In another study, cellulose sulfate edible films which were incorporated with β-cyclodextrins and mustard essential oil (MO) have been addressed. The MO addition increased the mechanical properties, however, it did not affect the water vapor permeability, but it showed a great antimicrobial activity against *E. coli* and *S. aureus*. A film-forming solution without MO showed antibacterial activity not only for the bacteria that are mentioned above but also against *Bacillus subtilis* and *Aspergillus niger*, thus proving the potential of cellulose sulfate’s properties. Moreover, these films did not cause alterations in the foods’ organoleptic properties and taste [56].

#### 2.2.3. Cellulose Nitrate

Cellulose nitrate (CN) or nitrocellulose is considered to be the first semi-synthetic polymer in the plastic industry. It is produced by nitration where the cellulose hydroxyl groups are substituted by the nitrate groups. Its properties such as a high mechanical strength, a good solubility, and compatibility with plasticizers explain its use in the explosives, plastics, coating, and ink industries. These properties and applications depend on the degree of nitration [51]. The flexibility and dimensional stability of CN has led to its use in cinematographic films and photography. However, these types of films are flammable [57]. CN is sensitive to heat and light, and to combat these disadvantages and to guarantee their environmental safety, controlling the CN degradation is essential [58]. Nitrocellulose can also be used in the application of biosensors and chips that can detect different antibodies, proteins, serums, and other chemicals that affect the homeostasis of the body [59]. In addition, CN is versatile, and it is beneficial in other fields, including in water filtration, electricity generation, catalysis, and coatings [60,61,62].

In the food packaging field, the antioxidant activity of CN films with Shellac and olive leaf extract (OLE) and grape pomace extract (GPE) was assessed. The research points out the potential of these two natural extracts, OLE and GPE, as components for antioxidant packaging formulation. Moreover, cellulose nitrate showed that it retained the natural antioxidants more effectively than Shellac did. These films could represent a valuable tool for the effectiveness of antioxidant packaging and contribute to a longer shelf life and the characteristics related to the improvement of packaged food [63]. However, this cellulose derivative is not used very much because there are some doubts about its film’s safety in food packaging applications [64]. 

#### 2.2.4. Ethyl Cellulose

Ethyl cellulose (EC) is an ether that is produced by the reaction of alkali cellulose with ethyl chloride at 60 °C for several hours. The properties and applications of ethyl cellulose depend on the degree of etherification, the molecular weight, and the molecular uniformity. Solubility in most organic solvents, such as ethanol, methanol, or ethyl acetate, is typically achieved when a degree of substitution between 2.2 and 2.6 is present. In this way, EC is insoluble in water. It is a biodegradable and relatively low-cost polymer, it is not toxic, and it has an excellent film-forming capacity. Moreover, it is water resistant, presents barrier-forming characteristics, and it is strong at room temperature. [51,65,66]. This polymer has the potential to be used in biomedical applications, especially in drug delivery. A novel drug delivery system based on EC consisted of 3D-printed tablets with an internal scaffold structure for the release of ibuprofen. EC, ibuprofen, and other excipients were mixed and extruded into filaments, and then, they were printed into tablets. An optimized and completed drug release process was achieved within 24 h by adding a certain amount of release modifiers and by adjusting the fill pattern, fill density, and the shell thickness of the models [67]. It was also possible to produce nanofibers as potential wound dressing materials or fabricated scaffolds for bone tissue engineering [68,69]. 

EC films have demonstrated to have great potential to substitute conventional plasticized films in different fields. In food packaging, pure ethyl cellulose films cannot extend the shelf life of food, so they are blended with active agents to improve their properties. Li et al. suggested the application of konjac glucomannan and ethyl cellulose blended films for food packaging. This film presented a good moisture resistance, thermal stability, tensile strength, and an elongation at the break [70]. Capsaicin, a substance with good antioxidant and anti-inflammatory properties, was also added to an EC-based film as an antibacterial agent that can inhibit the activity of microorganisms on the surface of the film. The study demonstrated that capsaicin and EC interacted through the phenolic group in capsaicin and the hydroxyl group in EC, showing that this substance can participate in the formation of the film. The application of the capsaicin-EC film was efficient in retarding the bell pepper ripening process [71]. Another study reported novel poly(vinyl alcohol)/ethyl cellulose/tea polyphenol electrospun nanofibrous films as an active packaging material, which exhibited good physical properties and biological activity, and presented a good preservation effect on pork, by extending its shelf life by 3 days [72]. 

#### 2.2.5. Methylcellulose

Methylcellulose (MC) is a cellulose ether derivative that is considered to be Generally Recognized As Safe (GRAS) by the U.S. Food and Drug Administration (FDA) [73]. MC can be produced by reacting alkali cellulose with a methylating agent, such as methyl chloride or dimethyl sulfate [51]. MC is available in a wide range of molecular weights grades, but commercially, it is available almost exclusively in low molecular weights (~10,000 Da) [74]. MC presents emulsifier, adhesive, and thickening properties, is water-soluble (up to 55 °C), and exhibits thermal gelation properties at elevated temperatures. Its aqueous solutions are stable across different pH values (2–12) without there being apparent changes in the viscosity parameters. Commercial MC is available with a viscosity of 4–3500 mPas. It is not soluble, or it shows lower solubility in solvents such as acetones or alcohols, thus limiting the available drug delivery formulation methods. However, all of these properties depend on its degree of substitution, solution concentration, salt content, heating rate, and polymer molecular weight [74,75].

Methylcellulose is used as an excipient in pharmaceutical formulations, as a coating agent for drug delivery and as a viscosity-enhancing polymer. It is also used as a film-forming and as a food additive. Sahoo and their co-workers synthetized gold nanostructures using environmentally friendly methylcellulose as a stabilizer and removal-reductant hydrogen gas as a reducing agent that is considered to be helpful for biomedical and pharmaceutical applications and also for large-scale production in the industry [76]. Its polymer gelation property has been demonstrated in several studies. In one of them, a hyaluronan/MC hydrogel promoted cell survival in vivo and tissue repair, and it is a versatile delivery vehicle for controlled biomolecule delivery directly to the central nervous system (to the brain, spinal cord, and retina). This represents great opportunities in regenerative medicine [77]. To modify the hydrophilicity of MC, crosslinking can be performed with chemical agents, such as aldehydes or epoxy compounds. Glutaraldehyde is a crosslinker that is often used in MC films, however, it is not recommended that should be used in food packaging due to its toxicity [78]. Usually, this agent improved the films’ properties, such as the water vapor barrier properties and its mechanical strength. Additionally, fruit extracts have also been incorporated into MC films for the improvement of their antimicrobial and antioxidant activities. In this way, the MC films can be used to ensure the foods’ safety and quality [79,80]. Another study reported the importance of MC and hydroxypropyl methylcellulose (HPMC) as an edible coating/film to increase the fruit’s glossiness and extend the shelf life of horticultural crops. They delayed their ripening, decreased the rate of respiration, and presented barrier properties [81].

#### 2.2.6. Hydroxypropyl Methylcellulose

Hydroxypropyl methylcellulose (HPMC) is a cellulose ether that is formed by a based-catalyzed heterogeneous reaction of cellulose with methyl chloride and propylene oxide [74]. HPMC is soluble in water, biodegradable, cheap, transparent, odorless, flavorless, stable, oil-resistant, nontoxic, and biocompatible. Moreover, it presents a thermoplastic behavior, an uncharged nature, and a film-forming capability. HPMC has also received GRAS classification approval by the FDA, European Parliament and Council Directive (EU), and the Joint Expert Committee on Food Additives (JECFA) [82,83]. Because of these properties, HPMC has been extensively applied in the pharmaceutical field as a drug delivery matrix (film or gel) and in the food industry as a film former, emulsifier, stabilizer, or thickener. According to Tundisi et al., HPMC can be used in several ophthalmic drug formulations, such as eye drops, gels, inserts, and films, for different ocular diseases (dry eye state, age-related macular degeneration, diabetic retinopathy, retinal edema, conjunctivitis, glaucoma, and others) [83].

The HPMC polymer is appropriate to be used as a packaging material to extend the shelf life of fresh food due to its strong functional properties. However, HPMC films still require improvement regarding their water vapor properties as they are highly hydrophilic. For this, active compounds have been added to HPMC. For instance, glycerol and sorbitol are commonly used as plasticizers to improve the elasticity of the films. Other properties such as antioxidant, antimicrobial, gas permeability, and mechanical are enhanced by the addition of several compounds. Essential oils, organic and inorganic substances, extracts, lipids, or antioxidants are examples of compounds that can be used for the films’ improvement [82]. Among the active compounds, curcumin is an example that presents antioxidant, anti-inflammatory, anti-carcinogenic, antitumor, and antimicrobial activities. Nascimento da Silva et al. were the first to report the effects of curcumin on the physical and morphological properties of HPMC films. They highlighted that curcumin altered the optical properties of the films and can be released from the film to the food [84]. Lee et al. prepared an HPMC film, incorporating oregano essential oil, and this showed excellent antibacterial effects, especially against *Salmonella typhimurium*, antioxidant properties, and improved UV and water vapor barrier properties, which could be useful in active packaging [85]. Vieira et al. prepared a film with HPMC and silver nanoparticles to extend the papaya’s shelf life by controlling the development of anthracnose since the films presented antifungal properties against *Colletotrichum gloeosporioides* [86]. Song et al. prepared a film that used HPMC, chitosan, and glycerol, which showed antibacterial activity against *E. coli* and *S. aureus*, and so it is a promising material for food packaging as well as wound dressing and in cosmetics applications [87].

#### 2.2.7. Carboxymethyl Cellulose

Carboxymethyl cellulose (CMC) is an ether that is obtained by the reaction of cellulose with monochloroacetic acid, where the hydroxyl groups are substituted by the carboxymethyl groups in carbons 2, 3, and 6 of each glucose residue [51]. After the etherification process, a linear long polysaccharide backbone with the carboxyl and hydroxyl surface groups is obtained. It has unique features such as a high chemical reactivity, water solubility, gelation capability, hydrophilicity, stability, non-toxicity, biocompatibility, and biodegradability. 

These properties depend on its molecular weight, the degree of substitution, and the distribution of the carboxymethyl substituents along the polymer chains, and due to this, CMC can be used as an effective viscosity-increasing agent, a rheological control agent, a binder, a stabilizer, and a film former. Some disadvantages are its insolubility in some organic solvents such as ethanol [75]. 

Similarly, to the other cellulose derivatives, CMC can be applied in different fields such as in the biomedical, pharmaceutical, textile, construction, food, plastics, cosmetics, paper, and oil industries. In the biomedical fields, CMC is used in tissue engineering [88,89], bone-tissue engineering [90,91], and wound dressing [92,93]. CMC films have also gained interest over the last years in pharmaceutical applications, specifically, in drug delivery [94]. 

For instance, Koneru et al., studied the effect of incorporating grapefruit seed extract in hydrogel NaCMC-HPMC films, and those showed an excellent antimicrobial activity [95]. In food products and their packaging, CMC has been widely used as an auxiliary agent due to some of its properties, such as it being odorless, tasteless, and noncaloric. When a plant oil was incorporated in carboxymethyl cellulose/bacterial cellulose/glycerol-edible films the spoilage, and the weight loss of oranges and tomatoes was prevented, and their shelf life was increased at various temperatures. Moreover, there were no changes in its odor or color for up to 9 weeks [96]. Riahi et al. prepared a CMC-based film with chitosan-based carbon quantum dots, and the mechanical properties of the film were improved without them interfering with the film’s transparency. Additionally, the film showed excellent antimicrobial and antioxidant properties, by inhibiting mold and bacterial growth on the lemons and extending the shelf life [97]. In addition to active films, CMC has also been used in intelligent films. You et al. prepared a konjac glucomannan/CMC/blackcurrant anthocyanin film that was pH-responsive, and it was capable of monitoring the freshness of the fish in real-time. Blackcurrant is one of the most abundant sources of natural anthocyanins, and it has the same pH-responsive activity as other anthocyanins. In the pH range of 2–12, the color of the blackcurrant solution changes from red to white, and then, it changes to yellow-green. In addition, it showed antioxidant, antibacterial, antiviral, and anticancer activities [98].

#### 2.2.8. Hydroxyethyl Cellulose

Hydroxyethyl cellulose (HEC) is produced by the treatment of pure cellulose (cellulose pulp) with sodium hydroxide solution to convert the cellulose into active alkali cellulose. This alkali cellulose reacts with gaseous ethylene oxide, and then, several etherification reactions occur, in which each hydrogen atom in the cellulose hydroxyl group is replaced by the hydroxyethyl group. This substitution is responsible for the polymer’s solubility in water [99]. HEC is a low-cost, tasteless, colorless light yellowish, and odorless powder. It is water-soluble, biocompatible, biodegradable, and nonionic, and it has the capability of thickening, binding, emulsifying, suspending, dispersing, stabilizing, and the ability to retain water and form films that provide good protective action. It is easily soluble in either hot or cold-water, producing solutions with a wide range of viscosities [100]. 

HEC is applied in different industrial fields, such as for thickening paints, the finishing of textiles, a thickener in cement mortar, and a sizing agent in paper making [101]. Ayouch et al. described the production of CMC-HEC hydrogel films using citric acid as a non-toxic crosslinking agent for its potential application in wastewater treatment. The films demonstrated a good thermal stability, high transparency, excellent swelling ability in neutral and in acidic media, and efficient adsorption for the removal of heavy metals and dyes from contaminated water sources [102]. In the biomedical field, HEC hydrogels have been prepared by the casting method technique and functionalized with tungsten oxide. They exhibit antimicrobial activity against Gram-negative and Gram-positive strains, and thus, they can be applied as a promising wound dressing material [103].

Regarding the food industry, several studies demonstrated the potential of HEC film in food packaging. Liu et al. produced an edible coating with HEC, sodium alginate, and asparagus extract for it to be applied on fresh strawberries during its storage. It showed the growth inhibition of *Penicillium italicum*, extending the postharvest life of the strawberries [104]. Fawal et al. considered that HEC films with zinc oxide are a promising material for food packaging applications due to their mechanical properties and antimicrobial activity [105]. In another study, a sustainable κ-carrageenan/HEC film reinforced with silica and silver nanoparticles showed significant activity against six common food pathogens, *S. aureus*, *Bacillus cereus*, *Listeria monocytogenes*, *B. subtilis*, *S. typhi*, and *Cronobacter sakazakii*. Thus, this film could be a potential candidate for active packaging and other biomedical applications [106]. 

#### 2.2.9. Hydroxypropyl Cellulose

Hydroxypropyl cellulose (HPC) is prepared by reacting alkali cellulose with propylene oxide at an elevated temperature and pressure, thus leading to a nucleophilic ring opening. HPC is a non-ionic, amphiphilic cellulose ether that is soluble in aqueous and polar organic solvents such as methyl alcohol, ethyl alcohol, and isopropyl alcohol (95%). It is soluble in water that is below 40 °C and has interesting thermal gelation properties such as MC and some other cellulose ethers. As a result of this gelation property, HPC is insoluble in water that is above 45 °C, it is thermoplastic, has emulsifying and thickening properties, and is a good film former [74,101]. 

HPC is applied in the biomedical and pharmaceuticals fields and as a food additive. In the biomedical field, the most important application is its use in ophthalmology as a lubricant for artificial tears, and thus, to treat medical disorders such as insufficient tear production due to corneal erosions, decreased corneal sensitivity, and neuroparalytic keratitis [107]. An example of an eye lubricant is LACRISERT^®^ (Lakewood, NJ, USA). Regarding the powder formulations, the effect of HPC on the nasal drug absorption of the powder was studied, and it was concluded that the nasal retention of different formulations was enhanced by HPC. This study indicated that the powder formulations that are supplemented with HPC are a promising approach to increase the nasal absorption of highly soluble and poorly permeable drugs [108]. Takeuchi et al. assessed the applicability of HPC to orally disintegrating film (ODF) and investigated the optimization of the formulation of HPC. Active pharmaceutical ingredients and excipients, such as ibuprofen and calcium carbonate, were added to the HPC formulation. The results showed that the disintegration time decreased as the thickness of the HPC films decreased, which can be controlled by the ingredients that were added. The HPC film with ibuprofen prolonged the disintegration time, and addition of the calcium carbonate particles to the film shortened it. Thus, HPC can be used as a base material for ODF applications [109].

In the food packaging field, the ability to formulate HPC gels from only water and “food-grade” constituents allow for a wide range of potential applications, ranging from mechanochromic materials, colorant-free food decoration to short-term sensors in biodegradable smart labels [110]. In another study, an HPC film incorporating rice straw oxidized cellulose nanocrystals (OXCNC) was used as a paper coating. The authors reported that the adding of OXCNC increased the water vapor permeability of the HPC films due to their hydrophilic nature, and that the HPC/10% OXCNC film was found to have the maximum tensile strength properties, and so, it was chosen for coating the bagasse paper sheets. This coating with HPC/OXCNC improved the mechanical properties and reduced the porosity more than the pristine HPC could [111]. 

To conclude, cellulose-based films are widely used in the food packaging industry because of their unique properties. With scientific advances, innovative approaches are increasingly being taken such as active and intelligent packaging, and those will be further discussed.

### 2.3. Novel Cellulose-Based Films Aiming Food Packaging Applications

Although cellulose is one of the most used biopolymers, cellulose-based films have some limitations, such as their moisture-barrier ability due to the hydrophilic nature of these films, and they have limited functional characteristics [27]. In the case of MC and HPMC, although they have a good resistance to carbon dioxide, oxygen, and lipid, they are poor water vapor barriers [112]. The use of CMC is also limited due to its poor mechanical properties. CA detains limited mechanical, barrier, and thermal properties [113]. Besides those of the cellulose-based films functionalization towards the improvement of their physicochemical properties, the addition of bioactive compounds may enhance their bioactive properties. Active packaging emerges as an innovative approach, promoting the improvement and reinforcement of the antioxidant and antimicrobial properties of the biodegradable films [114]. As a result, the reduction of the microbial contamination and spoilage resulting from active packaging materials not only extends the shelf-life, but it also improves the safety, quality, and even the sensory properties of the packed product. Some examples of the main compounds for the active packaging are oxygen, carbon dioxide, and ethylene scavengers; carbon dioxide emitters; odor emitters and absorbers; relative humidity regulators; antibacterial substances; antioxidants [115].

#### 2.3.1. Cellulose Films with Antimicrobial Properties

Antimicrobial food packaging is one of the most promising strategies. Bacteriocins, enzymes, metals, chemicals, and biopolymers are examples of active compounds that have been incorporated into packaging films, and they are shown in Table 1. These antimicrobial agents have been applied mostly in the packaging of fruits and vegetables, but also of fish, meat, and dairy products [116].

Since cellulose is an excellent biopolymer, its incorporation with active compounds will improve its properties. Several studies have demonstrated the antimicrobial properties of essential oils (EO) and plant extracts (PE) against a wide range of foodborne pathogens. EOs are organic substances that are obtained from leaves, flowers, seeds, buds, bark, and stems, and their antimicrobial effects are associated with the hydrophobic nature that allows them to permeate through membranes. PEs result from crude mixtures of natural compounds [117,118]. For instance, cellulose acetate films with the incorporation of pink pepper essential oil have demonstrated an antibacterial activity against Gram-positive foodborne pathogens (*Listeria monocytogenes* and *Staphylococcus aureus*) in sliced cheese, thereby extending its shelf life [119]. Another study was based on two essential oils, *Rosemary* and *Aloe Vera*, which were incorporated into the cellulose acetate films. The antimicrobial activity against Gram-negative (*E. coli*) and Gram-positive (*B. subtilis*) bacteria increased with the increase in the essential oil content [120]. Carboxymethyl cellulose films are also widely studied in the food packaging field. Sodium alginate/CMC films which were incorporated with cinnamon essential oil (CEO) demonstrated an excellent antimicrobial activity against *E. coli* and *S. aureus*, and the inhibitory effects on *S. aureus* increased with the increasing of the CEO. Furthermore, the CEO improved the physical properties of films, its incorporation increased the thickness, water vapor, and oxygen permeability and reduced the moisture content and tensile strength of them. These films can be used for the preservation of bananas, thereby extending their shelf life [121]. Additionally, with cinnamon oil, Noshirvani et al. prepared CMC/chitosan films. They also included ginger oil, and the results showed that the cinnamon oil plasticized the films and presented a higher antifungal activity in vitro against *Aspergillus niger* than ginger oil did [122]. Carboxymethyl cellulose–polyvinyl alcohol films with clove oil have been demonstrated to be active against *S. aureus*, and *B. cereus*. Ground chicken meat that had been packaged in these films showed lower total viable counts and displayed a shelf life of 12 days, whereas the control samples became spoiled within 4 days in the fridge [123]. For the preservation of high-fat meat products, a multifunctional CMC film that was combined with zinc oxide nanoparticles and grape seed extract was fabricated by Priyadarshi et al. [124]. The films showed antimicrobial activity against the common foodborne pathogens, *E. coli* and *L. monocytogenes*. Choi et al. tested the effect of a hydroxypropyl methylcellulose (HPMC) edible film containing oregano essential oil (OEO) and bergamot essential oil (BEO) at different concentrations for fresh “Formosa” plums. The antimicrobial activity of the films was tested against *E. coli,* and the results indicated that the *E. coli* colonies decreased with increasing essential oil concentration. However, the film containing OEO proved to be the most efficient regarding the antimicrobial effect and thus, it contributed to extending the plums’ freshness [125]. 

Bacteriocins are ribosomal synthesized antimicrobial peptides or complex proteins that are secreted by bacteria, which can kill or inhibit different bacterial strains. Because of their physical stability and non-toxicity, they have been reported to be food preservatives [126]. Nisin is the most common bacteriocin, and it is noted as being GRAS. Hence, several studies with cellulose derivatives, such as HPMC or CMC, have used this natural compound [127,128]. Bioactive films HPMC, nisin (N), and different percentages (from 5 to 75 wt.%) of TEMPO-oxidized nanofibrillated cellulose (NFC) that was isolated from rice straw pulp were prepared, and their properties were studied. The HPMC/N, NFC/N, and HPMC/N/NFC films exhibited significant antimicrobial activities against *S. aureus* with the visible controlled release of nisin in the case of films HPMC/NFC [127]. Freitas et al. prepared HPMC films with the incorporation of nisin Z under different concentrations (0%, 5%, 10%, 15%, and 20% wt.%), which is the most common variant of nisin that is used in the industry and in food research. The films with 10% (wt.%) of nisin Z and the control films were placed in contact with sliced mozzarella cheese for 8 days. The antimicrobial effect of nisin Z was verified against *S.* aureus and *Listeria innocua*. Nisin Z did not influence the mechanical properties of the films, and it is shows promise to be used for food packaging [128]. Lactic acid bacteria are also used as a food preservative as they present probiotic effects, show good antibacterial activity, and because the lactic acid that is produced can inhibit the growth of other bacteria. Lan et al. prepared edible films by embedding *Lactococcus lactis* in corn starch, and CMC. *L. lactis* improved the barrier properties of the films, and its antimicrobial activity against *S. aureus* prevailed after 8 days. These films can release 3.35 mg/mL of nisin since the *L. lactis* subspecies can produce this bacteriocin [129]. 

According to the World Health Organization (WHO), lysozyme (Lys) is considered a food preservative due to its antibacterial function and its stability at different pH and temperature values. It is a lytic enzyme that exists in animals, plants, and egg white, and its antibacterial principle is to catalyze the hydrolysis of 1,4-β-linkages glycosidic bonds between the N-acetylmuramic acid and N-acetyl-d-glucosamine residues that are present in the peptidoglycan layer of bacterial cells, thus leading to the damage of the Gram-positive bacteria cell walls [130,131]. In recent years, some studies have demonstrated the efficiency of lysozyme as an antibacterial agent in cellulose film preparation. In Hu et al.’s work, the presence of lysozyme improved the mechanical properties of the CMC films and showed an inhibitory effect against *S. aureus* and *E. coli* O157:H7, this presenting a better result against *S. aureus*, which can be explained by the differences in the cell wall structures of these bacteria [131]. Another study showed that gelatin/sodium CMC mucoadhesive films with lysozyme had a 100% bactericidal effect against *S. aureus* ATCC 25923 F-49, and they demonstrated activity against *Pseudomonas aeruginosa 415*, *E. coli* 055 K59912/4, and *Candida albicans* ATCC 855–653, which was somewhat smaller than the former, but it was still high [132].

Metal ions are capable of penetrating bacteria, inactivating their enzymes, or generating hydrogen peroxide, thus killing the bacteria [126]. Silver is one of the metals that is very often used as an antimicrobial agent. Marrez et al., reported that cellulose acetate film containing silver nanoparticles showed high antibacterial activity against *S. aureus*, *B. cereus*, *S. typhi*, *E. coli*, *K. pnuemoniae*, and low activity against *Pseudomonas* spp. In addition, the films showed no toxic effect on the larvae, thereby increasing the possibility of applying these films to active packaging [133]. Dairi et al. prepared a CA/triethyl citrate film with silver nanoparticles (AgNPs)/gelatin-modified montmorillonite nanofiller (AgM) and thymol (Th). The AgNPs influenced the antimicrobial activity, and *E. coli* was found to be most sensitive type to it. The results of this report showed great potential for CA/silver film applications to the active packaging of different food products [134]. In addition to silver, zinc oxide and copper oxide have also been used in cellulose films as an antibacterial agent. Ebrahimi et al. showed the preparation of CMC films with these three metals, demonstrating their activity against *E. coli* and *S. aureus* [113]. Another metal that can be used is titanium, which improves the mechanical, thermal, antimicrobial, UV-shielding, and water barrier properties of the films [135]. 

Chemicals such as sorbic acid have been used as preservatives in the food sector due to their efficacy in the weak acid pH range and their neutral taste. Sorbic acid is lipophilic, and it diffuses across the cell membrane and inhibits microbial growth. Yu et al., used potassium sorbate in methoxyl pectin/CMC films, and they observed that the release of the antimicrobial agent onto the food surface could contribute to food safety [136].

Some biopolymers are commonly blended with cellulose. Some of them, such as chitosan, are inherently antimicrobial. The antimicrobial properties of chitosan-CMC films were tested, and the shelf life of sliced bread increased from 3 to 35 days [137]. Other chitosan-CMC films were also investigated using zinc oxide nanoparticles. The prepared bionanocomposites displayed good antibacterial activity against Gram-positive (*S. aureus*) bacteria, Gram-negative (*P. aeruginosa*, *E. coli*) bacteria, and fungi (*C. albicans*). Moreover, these packaging films aided in increasing the shelf life of soft white cheese [138]. Other findings demonstrate that CMC-sodium caseinate films containing probiotic bacteria inhibit the growth of bacteria, thereby increasing the shelf-life of trout fillets, and maintaining their freshness for 14 days of storage [139].

**Table 1:** Summary of antimicrobial cellulose films functionalized with active compounds/extracts, showing beneficial properties for food packaging.

Active Compounds/Extracts	Cellulose Matrix	Functions	Ref.
Pink pepper EO	CA	- Antibacterial activity against Gram-positive foodborne pathogens (*L. monocytogenes* and *S. aureus*) in sliced cheese, thereby extending its shelf life.	[119]
*Rosemary* and *Aloe Vera* EOs	CA	- Antimicrobial activity against Gram-negative (*E. coli*) and Gram-positive (*B. subtilis*) bacteria.	[120]
Cinnamon EO	CMC	- Good activity against *E. coli* and *S. aureus*, thereby extending the shelf life of bananas.	[121]
Cinnamon and ginger EOs	CMC/chitosan	- Films with cinnamon EO presented a higher antifungal activity against *A. niger* than those with ginger EO; - Cinnamon oil plasticized films; - The films can be used for food preservation.	[122]
Clove oil	CMC	- Ground chicken meat samples packed in these films had lower total viable counts and displayed a shelf life of 12 days, whereas control samples spoiled within 4 days during refrigerated storage. Efficacy against *S. aureus* and *B. cereus* bacteria.	[123]
Zinc oxide nanoparticles/Grape seed extract	CMC	- Films displayed the potential to extend the shelf life of high-fat meats; - Potent antibacterial activity against *E. coli* and *L. monocytogenes.*	[124]
Oregano and bergamot EOs	HPMC	- The HPMC film containing 2% OEO had better antimicrobial properties against *E. coli* than those with 2% BEO did; - These films contribute to extending the plum’s freshness.	[125]
Nisin	HPMC	- The HPMC/N, nanofibrillated cellulose (NFC)/N, and HPMC/N/NFC films exhibited significant antimicrobial activities against *S. aureus* with noticeable controlled release of nisin in the case of films containing HPMC/NFC.	[127]
	HPMC	- Nisin Z presented antimicrobial activity mainly against *L. innocua* and *S. aureus*; - The mechanical properties of the films were not altered by the bacteriocin; - These films can be used as active packaging for dairy foods since they showed good results with sliced mozzarella cheese.	[128]
Nisin and Lactococcus lactis	CMC/corn starch	- The composite film with 1.5% *L. lactis* showed the highest release of nisin and good antibacterial activity against *S. aureus* after 8 days.	[129]
Lysozyme	CMC	- CMC-lysozyme film was more water-resistant, had better mechanical properties, and showed antibacterial properties.	[131]
	Sodium-CMC/Gelatin	- The enzyme in films retained more than 95% of its initial activity after 3 years of storage; - Films have a 100% bactericidal effect on the *S. aureus.*	[132]
Silver nanoparticles	CA	- High antibacterial activity against *S. aureus*, *B. cereus*, *S. typhi*, *E. coli*, *K. pnuemoniae*, and low activity against *Pseudomonas* spp. The films showed no toxic effect on larvae;	[133]
		- Antimicrobial activity against *E. coli.*	[134]
Silver, zinc oxide, and copper oxide nanoparticles	CMC	- Antibacterial activity against *E. coli* and *S. aureus.*	[113]
Titanium	CMC	- Improve the mechanical, thermal, antimicrobial, UV-shielding, and water barrier properties.	[135]
Potassium sorbate	CMC/Pectin	- Enhancement of food safety due to antimicrobial property.	[136]
Chitosan/Zinc oxide nanoparticles	CMC	- This packaging material increases the shelf life of sliced wheat bread, thereby restricting its water loss and decreasing fungal growth; - Improvement of mechanical properties.	[137]
Chitosan/Zinc oxide nanoparticles	CMC	- The obtained bionanocomposite extends the shelf life of cheese during the storage period, thereby displaying good antibacterial activity against *S. aureus*, *Pseudomonas aeruginosa*, *E. coli* bacteria, and fungi *Candida albicans.*	[138]
Sodium caseinate	CMC/Sodium caseinate	- Probiotic (*Lactobacillus acidophilus*, *L. reuteri*, *L. casei*, *L. rhamnosus*, and *Bifidobacterium bifidum*) CMC-sodium caseinate films increased the shelf-life of trout fillets.	[139]

CA—cellulose acetate; CMC—carboxymethylcellulose; HPMC—hydroxypropyl methylcellulose; HEC—hydroxyethyl cellulose; MC—methylcellulose; EO—essential oil.

#### 2.3.2. Cellulose Films with Antioxidant Properties

Lipid oxidation is another cause of food spoilage. The oxidation of food products results in a decrease in the nutritional value of the food, and consequently, a decrease in the energy content, origins odors, and color change, which are important factors for the consumer [140]. To solve this problem, the approach of the addition of antioxidants to foods or food packaging has been adopted, and this is shown in Table 2.

Antioxidants can be synthetic or natural. Natural antioxidants such as tocopherol, plant extracts, and essential oils from herbs and spices have been gaining more attention because of the toxicity of the synthetic antioxidants, such as polyphenol, organophosphate, and thioester compounds [141]. An example of the various natural antioxidants that can be used in active biodegradable films are carotenoids, which are a class of compounds that are usually used as natural dyes. According to Assis et al. [142], the addition of the natural antioxidants norbixin, lycopene, and zeaxanthin demonstrated the potential of their application in the development of cellulose acetate films. The addition of those carotenoids improved physical, mechanical, and barrier properties,. Other studies with cellulose derivatives (CMC, HPMC, HEC, and MC) have described that the incorporation of fruits extracts, such as murta fruit and pomegranate seed, contributed to the antioxidant activity of the films due to the diverse phenolic compounds content [79,143]. There are many other studies with CMC derivatives because of its good film-forming properties. He et al. described a CMC-gelatin film that was functionalized with the antioxidants of bamboo leaves. The obtained films improved the light barrier properties and scavenging of the DPPH radicals with an increasing antioxidant content [144]. More natural antioxidants such as Chinese chives, α-tocopherol, curcumin, and epigallocatechin gallate were incorporated into carboxymethyl cellulose films. The results showed that they could be used as antioxidant agents [145,146,147,148]. Berry extracts are also widely used in functional cellulose films. For instance, the leaves and fruits of Maqui have been used in the treatment of sore throats, kidney pains, ulcers, fever, lesions, migraines, etc. Additionally, several reports have already proven that maqui extracts could act as a cardio protective, antioxidant, digestive, anti-inflammatory, and anti-migraine agents due to their high content of phenolic compounds [80]. An ethyl cellulose derivative was mixed with polydimethylsiloxane (PMDS) to form films, and clove essential oil was added, which increased the flexibility and antioxidant activity of the film. In addition, the film showed also antimicrobial properties, by reducing the biofilm formation on the films [149]. Methylcellulose films were developed containing *Lippia alba* extract and silver nanoparticles. This extract contained several phenolic compounds that are capable of biosynthesizing the metal nanoparticles. In this case, the presence of the silver nanoparticles increased the antioxidant activity of the MC films. In addition, the films presented an antimicrobial activity [150]. Another study that used MC as a biopolymer for film production incorporated chitosan ascorbate which presented an excellent antioxidant capacity and a great ability for blocking UV-Vis light. Chitosan ascorbate that is blending with MC aids to improve its insufficient mechanical strength, which is important for food packaging materials. So, these films demonstrated good physicochemical and antioxidant properties, thereby showing their potential for their use in food packaging [151].

**Table 2:** Summary of antioxidant cellulose films functionalized with active compounds/extracts, showing beneficial properties for food packaging.

Active Compounds/Extracts	Cellulose Matrix	Functions	Ref.
Carotenoids: norbixin, lycopene, zeaxanthin	CA	- Films with norbixin or lycopene showed higher properties (lower formation of oxidation products) during the storage of sunflower oil under controlled oxidation conditions when they were compared to zeaxanthin. High stability of these pigments.	[142]
Murta extract	MC-Glutaraldehyde (GA)	- Cross-linker GA aid to increase water resistance of films and other mechanical properties; - Antioxidant and antimicrobial activities improved with a decrease in GA concentration.	[79]
Pomegranate seed extract	CMC, HEC, HPMC, MC	- The extract caused an increase in the antioxidant power of these films.	[143]
Antioxidants of bamboo leaves	CMC	- The light barrier properties and scavenging rate of DPPH radicals were enhanced with the increase in AOB content.	[1^144^]
Chinese chives root extract	CMC	- The antioxidant activity of films is enhanced by the addition of extract, and it increases with the increasing concentration of it.	[145]
α-tocopherol	CMC-lecithin/CMC-Tween80	- Films showed antioxidant activity, and when lecithin is incorporated α–tocopherol is more stable, increasing its function.	[146]
Curcumin and Zinc oxide	CMC	- Film showed strong antioxidant and antibacterial activity, and the curcumin was responsible for antioxidant activity; - UV light barrier properties were improved, contributing also to preventing photooxidation.	[147]
Epigallocatechin gallate(EGCG)	Sodium alginate (SA)-CMC	- The release and DPPH radical scavenging assay of incorporating EGCG in SA-CMC films showed that the obtained SA-CMC films with high EGCG content could release EGCG slowly and had strong antioxidant activity in fatty foods.	[148]
Maqui extract	MC-Glutaraldehyde (GA)	- The extract is a potential source of antioxidants, but when GA concentration increases, phenolic compounds decrease.	[80]
Clove EO	EC	- The addition of clove oil showed antioxidant and antimicrobial properties in the films. Its presence also reduced biofilm formation.	[149]
*Lippia alba* extract and Silver nanoparticles	MC	- The films with silver nanoparticles showed the best antioxidant activity; - The metal nanoparticles improved the mechanical properties and antimicrobial activity; - These films are a good alternative for food packaging as they are able to prevent lipid oxidation and increase the shelf life of food products.	[150]
Chitosan ascorbate	MC	- Great antioxidant activity and the ability for blocking UV-Vis light.	[151]

CA—cellulose acetate; MC—methylcellulose; HPMC—hydroxypropyl methylcellulose; CMC—carboxymethylcellulose; HEC—hydroxyethyl cellulose; EC—ethyl cellulose.

#### 2.3.3. Intelligent Cellulose Films

Intelligent packaging is an emerging technique in the food packaging industry. According to the EFSA, “Intelligent packaging” can be defined as the use of “Intelligent food contact materials that monitor the condition of packaged food or the surrounding environment, for instance by providing information on the freshness of the food” [152].

The intelligent systems can be, for instance, incorporated or printed labels on the food packaging material which offers the possibility to check product quality, track critical items, and provide more detailed information throughout the food supply chain (storage, transport, distribution and sale) [153]. While the active package is linked to the release of the active components that interact with the food and extent the shelf-life of the product, intelligent packaging provides the information that is related to food quality and safety to manufacturers, retailers, and/or consumers [153].

Examples of intelligent food packaging include the use of indicators, sensors and data carriers. For instance, sensors that can communicate the journey of the product from the production until it is in the hands of the consumer, showing for instance the temperature conditions that the product has been submitted during its life cycle. Other examples include freshness indicators that can be visual (by tags with color modification) or they may require technology such as QR codes that can be read by a smartphone [154]. 

The indicators can report on the product or environment changes, for instance, those relating to temperature, pH, oxygen, freshness. The information that is provided can be visual, qualitative, or semiquantitative, and it is often associated with the color change of a tag. Among the most studied ones are the pH indicators that provide information on the pH alterations that can occur with specific reactions that are related to the product’s freshness (e.g., formation of acids or amines). Other examples include indicators of the carbon dioxide production that is related to the microorganism contamination, pathogen indicators that work by reacting with toxins through various chemical and immunochemical methods (e.g., *Escherichia coli* O157 specific), and hydrogen sulfite presence that is related to meat deterioration [153,155]. 

The use of films is the simplest and cheaper means for developing intelligent food packaging. They can be obtained by the sensors and film-forming materials [156]. 

Although the sensors for monitoring food quality can be incorporated into films, cellulose-based intelligent films that can be found in the literature aims to monitor food freshness through pH indicators (Table 3). These freshness indicators are used to provide information on the microbial growth and chemical changes that lead to pH alterations, thereby leading to qualitative information through visual color changes [157]. Most of them were obtained by a combination of the biopolymers with extracts from fruits or vegetables since the presence of the natural pigments anthocyanins makes pH alterations visible by color changes [158,159]. According to several studies [160,161], the potential of the red cabbage extract was verified through an analysis of its color changes under different pH conditions. In this way, the red cabbage extract works as a good natural colorant for its incorporation in cellulose acetate and carboxymethyl cellulose matrix films. Still, in another study, red cabbage extract acted as a pH indicator. The researchers produced HPMC and red cabbage extract films and tested them in different pH solutions (2, 4, 7, and 9). All of these films detected ammonia, and thus, they changed their color [162].

In turn, pyranoflavylium salts are anthocyanin-derived pigments that also can change color according to pH variations, and they have been described as being more stable than anthocyanins. Gomes et al. reported the incorporation of this pigment into cellulose acetate-based films with plasticizer glycerol to build up a colorimetric pH indicator as a smart label for monitoring the food’s freshness [163].

Studies with berry extracts, such as mulberry and barberry, which were incorporated into the films of hydroxypropyl methylcellulose and methylcellulose matrices, respectively, also demonstrated that they can be used as pH-sensitive color indicators, and thus, they allowed the films to monitor the fish’s freshness [164,165]. Jambolão (*Syzygium cumini*) fruit also presents a great quantity of anthocyanins, which present color changing properties when they are under pH variations. MC films that were incorporated with Jambolão extract were produced, showing them to be pH-sensitive and that they presented an antioxidant activity. Thus, the produced films offer benefits by increasing the food’s shelf life and providing information on the product’s freshness to the consumers. Moreover, they were suitable to be applied in meat or aquatic products because these are the products wherein lipid oxidation occurs [166].

There are a few studies of blackcurrant anthocyanins, but they have the same response as other anthocyanins do [98]. Liu et al. fabricated a new pH-indicating film based on CMC and gelatin, curcumin, and chitosan for monitoring the freshness of pork [167]. Boonsiriwit et al. prepared an HPMC/microcrystalline cellulose film, incorporating butterfly pea anthocyanins that showed a high sensitivity to the pH and ammonia. These films showed to be promising in monitoring the fish’s freshness [168]. Jiang et al. prepared CMC/starch and purple sweet potato anthocyanins films, which were sensitive to ammonia and pH changes and can be used to monitor the real-time freshness of fish [169]. Moreover, CMC-ovalbumin films that were incorporated with blueberry anthocyanins were prepared to monitor the mushrooms’ freshness. Those films had a color response to carbon dioxide gas release and to different pH buffers, with it changing from a purple to a pink color [170].

## 3. Conclusions

The current review addresses the recent advances in biodegradable cellulose-based films for their diverse applications, and specifically, for food packaging that has been essential in recent decades. Different cellulose derivatives can be pointed out as alternatives for the petroleum-based materials that are used in food packaging due to their film-forming ability and biodegradability. The most common cellulose derivatives that are presented and discussed herein were cellulose acetate, cellulose sulfate, cellulose nitrate, ethyl cellulose, methylcellulose, hydroxypropyl methylcellulose, carboxymethyl cellulose, hydroxyethyl cellulose, and hydroxypropyl cellulose. The functionalization of cellulose-based films to be used in active packaging has also emerged since the incorporation of active molecules that will be released to the food and food environment may enhance the shelf life of the products and reduce the waste. Cellulose-based active films have been produced mainly by the incorporation of natural antimicrobials and antioxidants, and most of them were isolated from fruits, vegetables, or plants. On the other hand, the achievement of intelligent films with cellulose derivatives has only been explored as pH-sensitive films that can provide information on the pH variations that are often associated with reactions that occur as the product deteriorates. Thus, the present work has reviewed the most significant results that are related to active and intelligent packaging. All of these functionalized films for packaging have a low-cost, are safe, and easy to fabricate, and so, they are expected to be used in a near future due to their contribution to shelf-life extension, quality, and the safety of the food products.

Further research in this area of smart packaging such as combining active and intelligent solutions will certainly improve the packaging as it is known today.

## Figures and Tables

**Table 3:** Summary of pH-sensitive cellulose films.

Sensors	Cellulose Matrix	Functions	Ref.
Red cabbage extract	CA	- The films promoted a color-changing capacity, thereby showing an excellent ability to detect volatile ammonia due to food degradation.	[160]
	Sodium-CMC/*Artemisia sphaerocephala* Krasch gum (ASKG)	- The films responded to buffer solutions (from rose-bengal to aquamarine) and NH3 (from yellow-green to yellow).	[161]
	HPMC	- The films detected volatile ammonia and different color reversibility; - At pH 2, they did not show complete color reversibility, thereby indicating a possible application as an irreversible food spoilage sensor.	[162]
Pyranoflavylium salt	CA/Glycerol	- The films showed relevant and remarkable color changes at the pH range of food spoilage.	[163]
Mulberry extract	HPMC/konjac glucomannan	- The composite film allows real-time visual monitoring of fish freshness, and it changed from purple to gray to yellow as the freshness of the fish decreased.	[164]
Barberry extract	MC/Chitin nanofibers	- The films changed color from reddish/crimson to pink to yellow in response to the loss of fish fillet freshness.	[165]
Jambolão skins extract	MC	- The MC films were active, intelligent, and biodegradable. They presented pH-sensitivity and antioxidant activity; - The films can be used to increase shelf life and indicate freshness of meat and aquatic products.	[166]
Blackcurrant anthocyanins	CMC/konjac glucomannan	- The pH-responsive film can monitor the freshness of fish in real-time; - The anthocyanins contribute to the improvement of antibacterial and antioxidant properties of the film.	[98]
Curcumin	CMC	- The films could monitor the freshness of pork because the curcumin was pH-sensitive. The films change from yellow to dark yellow to light yellow.	[167]
Butterfly pea anthocyanin	HPMC/microcrystalline cellulose	- Physical and chemical properties were improved; - The films were sensitivity to changes in pH of mackerel (Scomber scombrus) and to ammonia.	[168]
Purple sweet potato anthocyanins	CMC	- The indicator films were sensitive to ammonia and pH changes, and they changed from red to blue and green when the fish became spoiled; - The films can be used to monitor the real-time fish freshness.	[169]
Blueberry anthocyanins	CMC/ovalbumin	- The intelligent film was used as mushroom spoilage indicator; - The films changed from purple to pink during the storage of mushrooms.	[170]

CA—cellulose acetate; CMC—carboxymethyl cellulose; HPMC—hydroxymethyl cellulose; MC—methylcellulose.
